# Assessment of Iatrogenic Damage to Adjacent Teeth After Applying Different Prevention Methods: A Cross-Sectional Study

**DOI:** 10.7759/cureus.72632

**Published:** 2024-10-29

**Authors:** Yara Faour, Dajma Abed, Nabil Alhouri

**Affiliations:** 1 Department of Fixed Prosthodontics, Damascus University, Damascus, SYR; 2 Department of Pediatric Dentistry, Faculty of Dentistry, Damascus University, Damascus, SYR

**Keywords:** adjacent dental surfaces, cross-sectional study, dental students, full crown preparation, iatrogenic damage

## Abstract

Aim: This study aimed to assess the iatrogenic damage to adjacent dental surfaces when using different prevention methods.

Methods: Fifty gypsum casts were evaluated in this cross-sectional study, and a total of 50 posterior teeth that required full crown preparation to receive fixed prostheses were included in this study. The casts were assigned into two groups. In group A, the dental abutment was separated from adjacent teeth by a metallic matrix band at the distal surface and a wooden wedge to the mesial surface. In group B, the dental abutment was separated from adjacent teeth by a metallic matrix band at the mesial surface and a wooden wedge to the distal surface.

Results: A low degree of iatrogenic damage was detected in groups A and B. Groups A and B exhibited a low degree of damage to adjacent dental surfaces, with no significant difference between the two groups (p > 0.05).

Conclusion: Bringing students' attention to the importance of maintaining the integrity of adjacent dental structures during full crown preparations in addition to separating the dental abutment from adjacent teeth through prevention tools had the best results in reducing iatrogenic damage and preserving adjacent dental surfaces.

## Introduction

Preserving adjacent teeth and dental tissues during crown preparation is considered one of the most important principles in prosthodontics [1,2]. The prevalence of incidental damage to adjacent teeth during full crown preparations has reached 73% according to Moopnar et al. (1991) [3] and 97% according to Lababidi et al. (2013) [4].

A recent study reported that 77.8% of incidental damage to adjacent teeth was recorded during crown preparation fulfilled by undergraduate students [5].

The frequency and extent of iatrogenic damage depend on several factors, including clinical conditions and the skill and experience of the practitioner [6,7].

Damaging the enamel of adjacent teeth leads to surface roughness, increasing susceptibility to demineralization and acid permeability, and greater plaque formation; such damage increases carious susceptibility and gingival disease [8].

Reshaping and polishing the affected enamel surface does not help achieve the same morphological characteristics as the intact enamel surface. In contrast, it increases the risk of secondary caries because of residual furrows on the enamel surface, as the enamel surface has a higher fluoride concentration, and the affected enamel layers are more susceptible to plaque accumulation [9].

To avoid this complication, students in their early studying stages may tend to incline the bur, which leads to exaggeration in tapering and over-reduction of the crown. This excessive preparation can damage the retention of the final restoration, weaken the tooth structure, and affect the vitality of the pulp [10]. Thus, it is important to provide patients with adequate prostheses while maintaining the integrity of adjacent dental surfaces [10].

With the high prevalence of iatrogenic damage reported across the dental literature and the lack of studies that provide suggestions and methods to overcome this problem and decrease its incidence, this study aims to assess the iatrogenic damage to adjacent dental surfaces when using different protection methods.

## Materials and methods

Study design

This study was designed as a retrospective cross-sectional study. The sample size was calculated using G*Power 3.1 sample size calculator at an alpha error of 5% and a study power of 90%. The effect size was calculated based on a previous study [10]. A minimum sample size of 50 posterior teeth was needed to detect differences between the groups.

Sample selection

**Figure 1:**
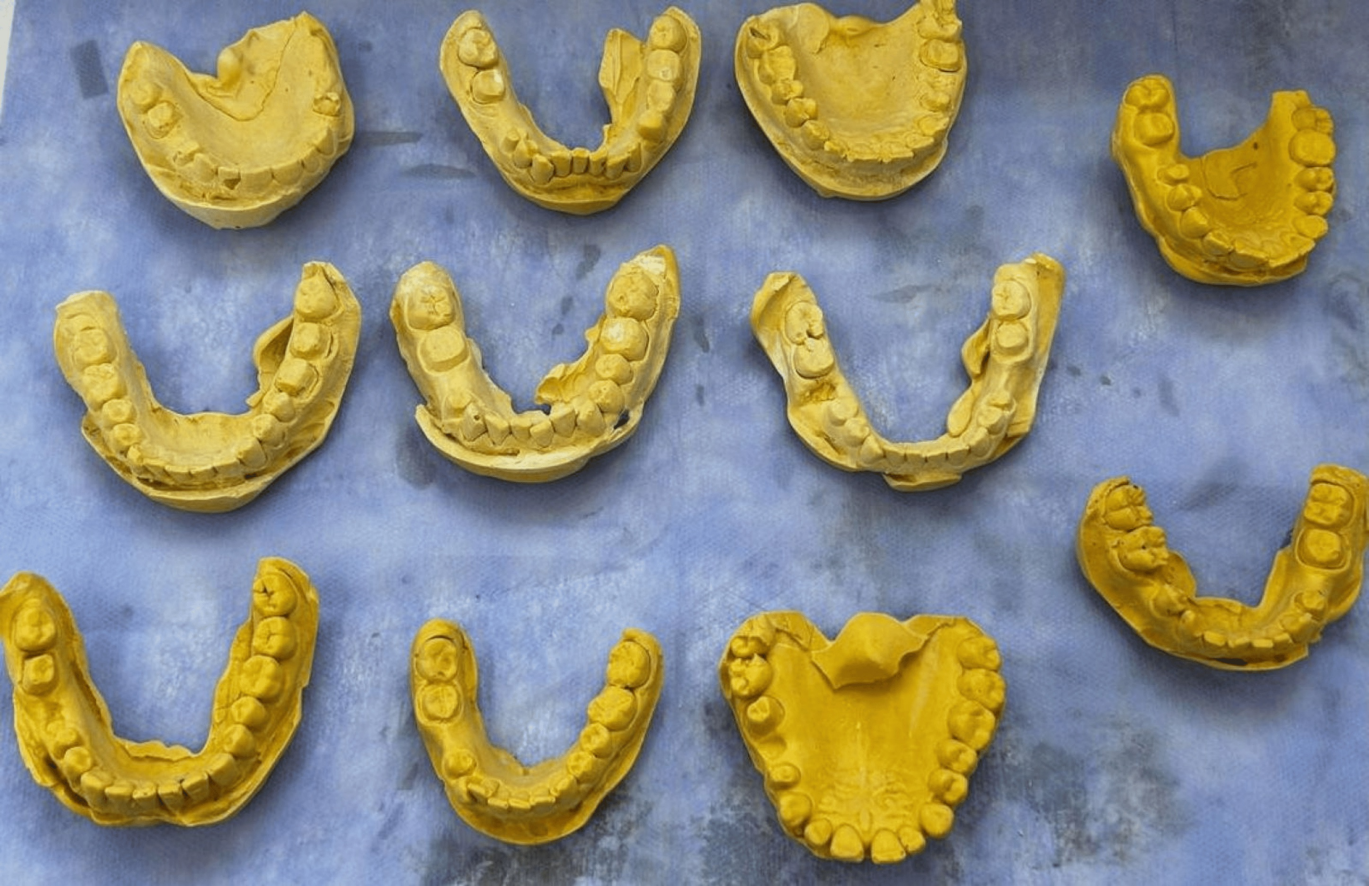
Some gypsum casts were included in the study.

A total of 200 gypsum casts of posterior teeth prepared by undergraduate students (fourth and fifth years) of the Faculty of Dentistry, Damascus University, were selected. These gypsum casts were divided into 100 gypsum casts of pre-prepared teeth and 100 casts of the same cases after preparation (final impression cast; Figure 1). Studied gypsum casts were chosen according to the following criteria: posterior teeth with intact adjacent teeth surfaces and posterior teeth that need to be clinically prepared to receive a full crown or a traditional metallic-ceramic bridge as part of the treatment plan.

**Figure 2:**
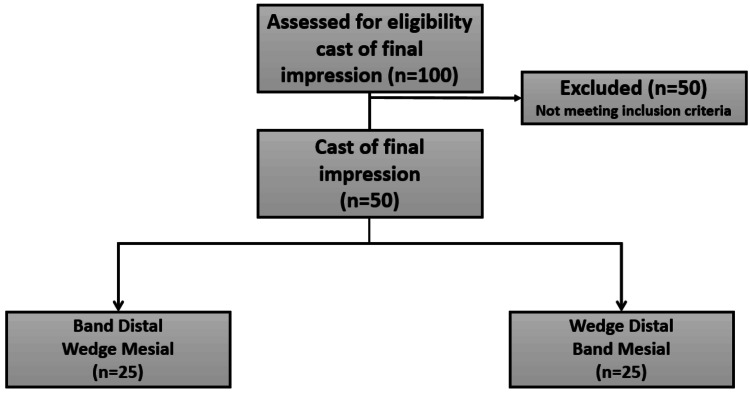
Sample selection diagram.

A total of 50 gypsum casts were eventually selected out of the 100 casts (final impression cast) according to the inclusion criteria (Figure 2), 25 gypsum casts were selected as the used protection method was a metal matrix band on the lateral side and a wooden wedge on the medial side, and the other 25 gypsum casts were selected as the used protection method was a wooden wedge in the lateral side and a metal matrix band in the medial side.

Therefore, the selected gypsum casts were divided into two groups: in group A, the protection method used is a metal matrix band on the lateral side and a wooden wedge on the medial side in 25 gypsum examples of prepared posterior teeth, and in group B, the protection method used is a wooden wedge in the lateral side and a metal matrix band in the medial side in 25 gypsum examples of prepared posterior teeth.

All these details are mentioned and documented in the archives of cases completed in the Department of Fixed Prosthodontics at the Faculty of Dentistry at Damascus University, and all cases included in the research were performed between 2021 and 2023, also, the protection methods used in the research are approved methods studied in the Department of Fixed Prosthodontics at Faculty of Dentistry in Damascus University, and these data are recorded in the department’s archive.

Outcome measurement

The gypsum casts were examined by an external evaluator with the naked eye and with the use of 2.5× magnifying loupes (Zumax, Jiangsu, China) under adjustable light and compared to the initial preoperational gypsum casts to verify the initial integrity of the adjacent surfaces and classify the degree of damage to both the mesial and distal surfaces according to the degree of damage (Table 1) [3].

**Table 1:** Degree of damage to both the mesial and distal surfaces according to Moopnar and Faulkner. Adapted from Moopnar and Faulkner (1999) [3].

Degree	Description
0	No damage visible to the naked eye or under a magnifying glass (x2.5)
1	Slight damage visible to the naked eye and identifiable with a magnifying glass
2	Obvious damage

Statistical analysis

Descriptive statistics were calculated using SPSS version 22 (IBM Corp., Armonk, NY) in terms of number, frequency, and percentages. The independent sample t-test was performed to detect any associations or significant differences in categorical variables (degree of damage) among the study groups.

## Results

A total of 50 gypsum casts with 50 posterior teeth that were in contact with at least one adjacent tooth surface and needed full crown preparation to receive a fixed prosthesis were included in this study. The score and percentage of iatrogenic damage to adjacent surfaces were recorded as follows (Table 2).

**Table 2:** Degree of damage” index score and percentages for mesial and distal surfaces in all four groups.

Variables			Group A	Group B
Degree of damage to distal surfaces	No damage	n (%)	12 (48%)	12 (48%)
	Slight damage	n (%)	9 (36%)	11 (44%)
	Obvious damage	n (%)	4 (16%)	2 (8%)
Degree of damage to mesial surfaces	No damage	n (%)	10 (40%)	13 (52%)
	Slight damage	n (%)	14 (56%)	11 (44%)
	Obvious damage	n (%)	1 (4%)	1 (4%)

The results showed that the mesial surface was intact in 12 (48%) in groups A and B, slight damage occurred in nine (36%) in group A and 11 (44%) in group B, and obvious damage occurred in four (16%) in group A and two (8%) in group B.

The results showed that the distal surface was intact in 10 (40%) in group A and 13 (52%) in group B, slight damage occurred in 14 (56%) in group A and 11 (44%) in group B, and obvious damage occurred in one (4%) in group A and one (4%) in group B.

Comparing the “degree of damage” index mean scores related to adjacent mesial surfaces

The paired sample t-test was applied for group comparisons. Groups A and B showed the least degree of damage to the adjacent mesial surface (p < 0.05), with no significant difference between them (P-value > 0.05) (Table 3).

**Table 3:** Results of independent sample t-test for study groups' comparison regarding the “degree of damage” data collected for adjacent mesial surfaces.

Compared groups	T-value	P-value
A	0.94	0.346
B		

Comparing the “degree of damage” index mean scores related to adjacent distal surfaces

The paired sample t-test was applied for group comparisons. Groups A and B showed the least degree of damage to the adjacent distal surface (p < 0.05), with no significant difference between them (P-value > 0.05) (Table 4).

**Table 4:** Results of independent sample t-test for study groups' comparison regarding the “degree of damage” data collected for adjacent distal surfaces.

Compared groups	T-value	P-value
A	0.567	0.570
B		

## Discussion

Iatrogenic damage is considered one of the most common problems that affect adjacent dental surfaces during full crown preparation of dental abutments [11]. The adverse effects of this type of damage on affected teeth include high susceptibility to dental caries, increased thermal sensitivity, and periodontal disease [12]. Thus, protecting the proximal surfaces of adjacent teeth during coronal preparation of dental abutments is considered one of the important biological principles in fixed prosthodontics and determines the criteria for successful coronal preparation and the quality and sustainability of dental prostheses [13].

Several studies have reported a high incidence of iatrogenic damage related to dental abutment coronal preparation; however, a review of the literature revealed that few to no studies have discussed or provided methods to reduce this incidence [14,15].

The results of the current study showed that there was no difference between the two protective methods used when preparing crowns, as the percentage of surface integrity was close in both methods.

The percentage of damage decreased when prevention methods were applied to separate the dental abutment from adjacent teeth. Students who were instructed to apply a metal matrix band and a wooden wedge at both the mesial and distal surfaces showed a low prevalence of obvious iatrogenic damage (16% to distal surfaces, 4% to mesial surfaces), with no difference between the two groups.

These results agree with Ballo's (2019) results, which reported a high percentage of iatrogenic damage when students did not apply any prevention tool during coronal preparation (92%), and the percentage decreased to 76% when students were instructed to apply a metallic matrix band. However, the overall percentage of damage remained high [10].

Similarly, Milic et al. (2015) reported a decrease in iatrogenic damage when a metallic matrix band or a wooden wedge was applied by dental students during class II cavity preparation to protect adjacent teeth (50% and 46%, respectively), whereas preparations that were not accompanied by any prevention tools showed a greater incidence of iatrogenic damage (74%) [14].

This study had some limitations such as the inability to apply more techniques to protect adjacent teeth and the inability to assess the development of students' abilities in the future.

## Conclusions

Due to the lack of expertise that undergraduate dental students have and because of the urgent need to minimize iatrogenic damage to adjacent dental surfaces during full crown preparation, this study shows that students should be frequently alerted on the importance of maintaining the integrity of adjacent dental structures, in addition to applying metallic matrix bonds and wooden wedges, which serve as effective methods for protecting adjacent teeth surfaces and reducing iatrogenic damage.
